# Editorial: Eating in the Age of Smartphones: The Good, the Bad, and the Neutral

**DOI:** 10.3389/fpsyg.2021.796899

**Published:** 2021-12-06

**Authors:** Jean C. J. Liu, David A. Ellis

**Affiliations:** ^1^Division of Social Sciences, Yale-NUS College, Singapore, Singapore; ^2^Neuroscience and Behavioral Disorders Programme, Duke-NUS Medical School, Singapore, Singapore; ^3^School of Management, University of Bath, Bath, United Kingdom

**Keywords:** smartphones, screen time, digital health, appetite, weight regulation, obesity

Worldwide, an estimated 6.4 billion individuals own a smartphone—a cell phone that provides communication and computing functions through an operating system (Statista, 2021a). The average smartphone user now engages with their device shortly after waking up and will spend 3–4 h a day interacting with a variety of apps (Andrews et al., 2015; Rootmetrics, 2018; Nielson, 2020). This frequent use, coupled with an ever-expanding range of social functions, means that devices are readily available throughout the day. Beyond being a potential distraction while eating, such functionality also allows for thoughts or food-related behaviors to be captured and shared (Teo et al.; La Marra et al.). Unlike single function screen-based technologies of the past, smartphones are not only changing how we live but also how we conduct research in the digital age. Therefore, we commissioned a Research Topic to understand how smartphones are transforming the eating experience.

Recognizing the timely nature of this topic, we invited researchers to specifically document “the good, the bad, and the neutral” aspects of eating in the age of smartphones. While a large body of research previously described how other devices (e.g., televisions) influence eating behaviors (Martin et al., 2009; Zhang et al., 2016), only a handful of studies before this collection focused directly on smartphones (e.g., Gonçalves et al., 2019; Yong et al., 2021). The curated collection responds by articulating how smartphones have taken their place in an obesogenic environment. While articles span both research papers and commentaries, each describes how smartphones might alter appetite regulation or increase the risk for weight gain. These collectively demonstrate that, as with the impact of other digital technologies, changes to eating behavior may not be unique to the technology itself. Instead, impacts can often be aligned with well-established psychological processes including social facilitation or attentional limitations, which may also disrupt the encoding of memories that affect appetite control (La Marra et al.).

In terms of research papers, Tebar et al. surveyed adults on their use of smartphones, televisions, and computers during the COVID-19 pandemic. Compared to participants whose phone use did not change throughout this period, those who reported greater phone use were 1.5 times more likely to report increased consumption of sweetened food (Tebar et al.). This pattern also occurred amongst participants who watched more television, but not for participants who reported increased computer use. In a separate paper, Lopez et al. examined the weight status of pre-adolescent children aged 9–11. Children were then asked about their propensity to multi-task using digital devices—for example, by checking their phones while completing their homework. In line with Tebar et al.'s findings, children who engaged more frequently in media multi-tasking were more likely to have a greater body mass index. Finally, Teo et al. conducted an experiment to examine how two forms of phone use would impact snacking behaviors amongst male adolescents. The authors found that when adolescents used their phones to send and receive messages, they consumed more snacks than when they used their phones to browse a neutral article.

While these papers provide empirical data across the lifespan, two further commentaries discussed possible mechanisms through which smartphones may influence our eating behaviors. At the individual level, La Marra et al. consider how smartphones may simply interfere with physiological signals of hunger and satiety. If someone is less aware of how much he or she has eaten, this could result in overeating, but this can occur with a wide variety of distractions (technological or otherwise) (e.g., Gonçalves et al., 2019). Even results that appear specific to key smartphone functions may simply reflect group processes observed offline. For example, previous studies have found that eating behaviors increase in the presence of other people and the virtual company offered by smartphones through messaging, video calling or other social networks may increase food consumption *via* a similar mechanism (Teo et al.). On the other hand, Stephens et al. argue that the convenience offered by phone apps alone may be a larger societal driver of unhealthy eating. Notably, phone-based food delivery apps have grown in popularity over the past decade. While some apps can support a healthy diet, most that offer delivery services disproportionately provide access to “junk” food and a frequent user's weight may increase as a result.

Taken together, the articles in our Research Topic highlight several ways in which smartphones may alter the eating experience. Two decades after the first smartphone became available to the public, research questions and methods continue to evolve. Eating can consume attentional resources, but it often occurs alongside a variety of evolving digital distractions. These innovations can initially make research more challenging. However, changes to everyday experiences following the adoption of smartphones provides many new opportunities for advancing psychological theory. Triangulating results from experimental and observational approaches could, for instance, inform theories of attention that have implications for daily life (La Marra et al.; Lavie, 2010).

Future research might specifically explore whether the impact of smartphones differs as a function of (i) the phone user (e.g., adolescents versus adults); or (ii) the type of phone use (e.g., passively watching videos versus actively playing games). However, given the variety of other biological, environmental, social, and cognitive factors that can also impact dietary behavior, the direction of any effect may not always be inherently obvious. For example, engaging with content that is very distracting could result in over *or* under-eating and this may vary between different groups. Interactions between individual differences and specific technology behaviors will, in turn, become even more important if new research is to capitalize on recent methodological advances (Ellis, 2020).

Most designs continue to rely on snapshots of self-report to capture both phone use and dietary behavior, however, smartphones can become part of a researcher's toolkit. Such approaches help mitigate methodological limitations and allow for longitudinal designs with larger sample sizes that capture dynamic patterns of behavior *via* experience sampling (e.g., Yong et al., 2021). For example, the ubiquitous nature of smartphones means that they can be used to track eating patterns directly by allowing participants to keep diaries or take photographs of their food (Ellis, 2020). Advances in image recognition that allow photos of food to be automatically classified will likely provide even more exciting opportunities for researchers in this area (Tran et al., 2020). However, even without such developments, smartphones can already be used to deliver or monitor the effectiveness of behavioral interventions (e.g., Piazza et al., 2021). As smartphone adoption is predicted to grow over the next decade (Statista, 2021b), we hope that this collection contributes to the conversation, spurring further research on what it means to eat in an age of smartphones.

## Author Contributions

Both authors co-wrote the editorial and approve of the final version.

## Conflict of Interest

The authors declare that the research was conducted in the absence of any commercial or financial relationships that could be construed as a potential conflict of interest.

## Publisher's Note

All claims expressed in this article are solely those of the authors and do not necessarily represent those of their affiliated organizations, or those of the publisher, the editors and the reviewers. Any product that may be evaluated in this article, or claim that may be made by its manufacturer, is not guaranteed or endorsed by the publisher.
